# The Death of the President

**Published:** 1881-10

**Authors:** 


### The Death of The President.

As we go to press, the City of Chicago is putting on the garb of mourning for the grievous loss it has sustained in the death of the Chief Magistrate of the Nation, JAMES A. GARFIELD. In common with the people of this and other countries, we feel keenly the grief which is awakened not only by the atrocious character of the wicked crime which has led to this result, nor yet only by the lofty position of the victim of the assassin's blow, but by the personal character of the noble martyr. His was a scholarly, an intellectual life, which appeals to the sympathies of all scholarly men, the world over. His was a gentle, home-loving and home-honoring life, which the heads of the families in this broad land were prompt to revere. His was a forgiving, unmurmuring and charitable spirit, such as that whose influence was evident in the counsels of the nation years after the body of Abraham Lincoln was laid in the dust. We have tears for his loss, gratitude for his example, reverence for the man who went down to the valley of the shadow of death without a reproach upon his lips for his murderer, and loyal honor for the gentleman who by this accident, becomes in his place the President of these United States.

One word as to the medical and surgical treatment of the case. Manifestly, until the professional gentlemen who have been in charge of it, shall have made up their record and presented it in full to their peers in medicine, it would be a discourtesy to either criticise or condemn. In the high position of Drs. Agnew and Hamilton, the profession at large have a guarantee that what was done was well done and worthily. The results of the autopsy have shown that no knowledge as to the course exactly traversed by the ball, was had by those who treated the wound. This is exactly and precisely the position taken from the first by Drs. Hamilton and Agnew. When questioned and interviewed, these gentlemen have repeatedly and properly asserted that it was impossible to state what course the ball had traversed. The newspapers, with characteristic savagery, are even at this moment assuming that a grave error had been committed. It will be remembered that at the time of the interesting and valuable investigations made by Dr. Weisse, of New York, some account of which has appeared in these pages, Dr. Hamilton was asked his opinion of Dr. Weisse's theory. The reply was, that he (Dr. Hamilton) was "interested," in the same. He could not, however, state that the course of the ball had been decided by the experiments, because it was his opinion that the former "had not been determined." As for the treatment of the suppurating track which many took to be the course of the ball, that surely should have been the same in either event. It is no small tribute to the skill and wise judgment of the surgeons of the late president, that they wisely refrained, in the face of considerable popular clamor, to seek for the missile to an extent which might have proved mischievous, and, as the result shows, would have proved futile; the fact being that now when the case is ended and its hopeless features evident to all, there can be but praise for the skill which ministered to the invalid for so long a period without once placing his life or comfort in jeopardy.

